# Putting the Spotlight Back on Plant Suspension Cultures

**DOI:** 10.3389/fpls.2016.00297

**Published:** 2016-03-11

**Authors:** Rita B. Santos, Rita Abranches, Rainer Fischer, Markus Sack, Tanja Holland

**Affiliations:** ^1^Plant Cell Biology Laboratory, Universidade Nova de Lisboa, Instituto de Tecnologia Química e Biológica António XavierOeiras, Portugal; ^2^Fraunhofer-Institut für Molekularbiologie und Angewandte Oekologie (IME), Integrated Production PlatformsAachen, Germany; ^3^Biology VII, Institute for Molecular Biotechnology, RWTH Aachen UniversityAachen, Germany

**Keywords:** plant suspension cultures, biopharmaceuticals, BY-2, protein production, plant cell cultures

## Abstract

Plant cell suspension cultures have several advantages that make them suitable for the production of recombinant proteins. They can be cultivated under aseptic conditions using classical fermentation technology, they are easy to scale-up for manufacturing, and the regulatory requirements are similar to those established for well-characterized production systems based on microbial and mammalian cells. It is therefore no surprise that taliglucerase alfa (Elelyso®)—the first licensed recombinant pharmaceutical protein derived from plants—is produced in plant cell suspension cultures. But despite this breakthrough, plant cells are still largely neglected compared to transgenic plants and the more recent plant-based transient expression systems. Here, we revisit plant cell suspension cultures and highlight recent developments in the field that show how the rise of plant cells parallels that of Chinese hamster ovary cells, currently the most widespread and successful manufacturing platform for biologics. These developments include medium optimization, process engineering, statistical experimental designs, scale-up/scale-down models, and process analytical technologies. Significant yield increases for diverse target proteins will encourage a gold rush to adopt plant cells as a platform technology, and the first indications of this breakthrough are already on the horizon.

## Introduction

Protein-based drugs are big business. The market for biopharmaceuticals is growing faster than the pharmaceuticals market as a whole, and a recent projection suggested the value of this segment could reach $US 278.2 billion by 2020 (PMR, 2015). There are more than 200 approved biopharmaceuticals on the market today and many more in the clinical pipeline (Walsh, 2014). Currently, most biologics are produced in microbes or mammalian cells growing in fermenters. Microbes are simple and inexpensive but often fail to produce complex proteins or those requiring specific post-translational modifications, whereas mammalian cells can achieve these folding and modification tasks with aplomb but only at a much higher cost. Both systems also have the potential for undesirable contaminants—endotoxins in the case of bacteria, and viruses or other pathogens in the case of mammalian cells. The extra steps required during downstream processing to remove these contaminants can increase production costs even further (BOX 1).

BOX 1 **COMPARISON OF MAJOR PRODUCTION PLATFORMS**

Industry platforms for the production of recombinant proteins are based mainly on microbes and mammalian cells. The major microbial system is the bacterium *Escherichia coli* which was the first species used to produce a recombinant human protein (somatostatin in 1977, Itakura et al., 1977) and the first to be used for the production of a commercial therapeutic protein (recombinant human insulin, approved in 1982 and marketed by Eli Lilly & Co. under license from Genentech). Many simple and unmodified proteins are produced commercially in *E. coli* but more complex proteins are difficult to fold unless targeted to the periplasm and this is not a scalable process (Baneyx and Mujacic, 2004; Choi and Lee, 2004). *E. coli* is simple and inexpensive but problems include the accumulation of proteins as insoluble inclusion bodies and the production of endotoxins that can cause septic shock. Yeasts are sometimes preferred because they share the advantages of bacteria but they are eukaryotes and thus support protein folding and modification, although the glycan chains are often longer than in mammals. *Saccharomyces cerevisiae* was the first yeast used to express recombinant proteins and it is still used commercially to produce a *Hepatitis B virus* vaccine, but other yeasts such as *Pichia pastoris* and *Hansenula polymorpha* are now favored during process development because they are more suitable for in-process inducible expression (Gerngross, 2004). Mammalian cells have dominated the biopharmaceutical industry since the 1990s because they can produce high titers (1–5 g/L) of complex proteins with mammalian glycan structures (Chu and Robinson, 2001). They are much more expensive than microbes but most pharmaceuticals are glycoproteins and the quality of the product is superior when mammalian cells are used. CHO cells are preferred by the industry but others that are widely used include the murine myeloma cells lines NS0 and SP2/0, BHK and HEK-293, and the human retinal line PER-C6. The major disadvantage of mammalian cells remains the cost of production, purification, and the risk of contamination with human pathogens.

The choice of expression hosts has more recently expanded to include plants because they offer unique features compared to the current dominant production systems (Stoger et al., 2014; Ma et al., 2015). The production of recombinant proteins in plants, where the protein itself is the desired product, is often described as *molecular farming*. If the proteins are pharmaceuticals then a bit of wordplay offers *molecular pharming* as an alternative. Plants combine the advantages of higher eukaryotic cells (efficient protein folding and post-translational modification) with the use of simple and inexpensive growth media. The diversity of molecular farming technologies is much greater than other production platforms, which can be advantageous or disadvantageous depending on the perspective (BOX 2).

BOX 2 **DIVERSITY OF MOLECULAR FARMING TECHNOLOGIES**

The immense diversity of molecular farming systems reflects the fact that recombinant proteins have been produced in many different plant species wherein there is a choice of whole plants or various cell/tissue culture formats (Twyman et al., 2003, 2005). Each of these may be suitable for stable expression (including nuclear and plastid transformation is some species) and transient expression (which can be achieved using *Agrobacterium tumefaciens*, plant virus vectors or combinations of both; Paul et al., 2013). Transgenic terrestrial plants are the most established platform and following a period of extensive diversification the field has now consolidated mainly to support tobacco as the primary leafy crop and the cereals maize, rice, and barley (Nandi et al., 2005; Tremblay et al., 2010; Sabalza et al., 2013). The main difference between these platforms is that leaves are watery tissues and the recombinant protein must be extracted quickly to avoid degradation whereas cereal seeds are desiccated and the protein remains stable for long periods. Cereal seeds are also suitable for direct oral administration. Aquatic plants such as duckweed and moss are also used as platforms (Reski et al., 2015). These have properties in common with terrestrial plants (differentiated whole plants) and cell suspension cultures (grown in containment in simple medium). The technology for aquatic plants and cell suspension cultures is similar but aquatic plants require light, whereas undifferentiated cell suspension cultures are grown in the dark but require a carbon source. After transgenic whole plants and cell suspension cultures, the third major technology platform is transient expression, which involves the introduction of non-integrating (episomal) vectors into leaves. The two main transient expression strategies are agroinfiltration, where leaves are infiltrated with *A. tumefaciens* by injection or vacuum leading to the transfection of millions of cells and the production of large amounts of recombinant protein in a short time (Komarova et al., 2010), and the use of recombinant plant viruses that infect cells directly, replicate within them and spread by cell-to-cell movement and systemic spreading through the vascular network to produce recombinant protein in every cell (Yusibov et al., 2006). A midway strategy that achieves biocontainment is the use of deconstructed virus genomes delivered by *A. tumefaciens*, which results in the transfection of many cells with the virus genome followed by its cell-to-cell movement but no systemic spreading (Peyret and Lomonossoff, 2015). All three major platforms have advantages and disadvantages—transgenic plants have a slow development cycle but are the most scalable, cell suspension cultures have a quick development cycle and allow contained production but are the least scalable, and transient expression allows the rapid production of high protein yields ideal for emergencies such as vaccines and prophylactic antibodies, as seen in the recent outbreak of Ebola virus disease in West Africa (Arntzen, 2015), but the large number of bacteria introduced into the leaves increases the endotoxin load (Arfi et al., 2015).

One niche of molecular farming technology that is now coming back into the limelight is the use of plant cells, specifically plant cell suspension cultures, rather than whole plants (Doran, 2000; Hellwig et al., 2004). Although molecular farming conjures up images of greenhouses bursting with dense green leaves containing valuable pharmaceutical proteins, much of the technical and commercial progress made in molecular farming has been based on plant cells. These combine the advantages of plants with those of traditional fermenter systems: contained, controlled and sterile production environments, chemically defined media lacking animal components, and compatibility with the toughest regulatory guidelines in existence—pharmaceutical good manufacturing practice (GMP). Recent advances in process engineering have seen plant cells leap forward toward commercial viability much faster than the established platforms achieved during their own development phases. The first molecular farming product approved for human use is manufactured in plant cells—and this is only the beginning (Zimran et al., 2011; Tekoah et al., 2015).

## Plant cell suspension cultures—platforms and products

The production of recombinant proteins in plant cell suspension cultures was first demonstrated more than 25 years ago (Sijmons et al., 1990) but progress over the subsequent decade was overshadowed by whole plants, and only a small number of studies involving cultivated plant cells as production hosts were published before the turn of the century (Table 1). The status of plant cells began to change after the first bubble of commercial interest in molecular farming collapsed due to the absence of a regulatory pathway, the opposition to GM crops (particularly in Europe), and the lack of support from an industry already heavily invested in fermenters. Whereas, some in the molecular farming community worked toward establishing regulations for pharmaceuticals derived from whole plants (Arfi et al., 2015; Ma et al., 2015; Sack et al., 2015) others realized that plant cells were already similar in many ways to microbial and mammalian cells and could be handled under the existing regulations (Ramachandra Rao and Ravishankar, 2002; Zimran et al., 2011).

**Table 1:** **Biopharmaceuticals produced in different plant cell suspension cultures**.

**Host cell**	**Variety**	**Protein**	**Indication**	**Yield**	**References**
Tobacco cells	BY2	Hepatitis B Surface Antigen (HBsAg)	Hepatitis B vaccine	6.5 μg/g FW	Smith et al., 2002
		PRX-102 (α-Galactosidase-A)	Fabry disease		Kizhner et al., 2015
		EPO	Tissue protective function	Low	Matsumoto et al., 1995; Pires et al., 2012
		Granulocyte-Macrophage Colony- Stimulating Factor (GM-CSF)	Production of white cells	Up to 250 μg/L	James et al., 2000; Lee et al., 2002
		IL-4	Immunoregulation	0.18 μg/L	Magnuson et al., 1998
		α-HBsAg Mab	Hepatitis B antibody	Up to 15 mg/ L	Yano et al., 2004; Sunil Kumar et al., 2007
		2G12 monoclonal α-HIV Ab	Anti-HIV antibody	12 mg/L SN	Holland et al., 2010
		Human Growth Hormone	Growth hormone	Up to 35 mg/L	Xu et al., 2010
		Human Interferon α2b	Anti-viral and immunomodulator	0.2–3% TSP	Xu et al., 2007
		IL-10	Immunoregulation	Up to 3% TSP	Kaldis et al., 2013
		Norwalk virus capsid protein	Acute gastroenteritis vaccine	Up to 1.2% TSP	Zhang and Mason, 2006
		IL-12	Immunoregulation	Up to 160 μg/L	Kwon et al., 2003b
Rice cells	*Oryza sativa*	Human α1-antitrypsin	Emphysema	4.5–7.7 mg/L Up to 150 mg/L	McDonald et al., 2005; Trexler et al., 2005
		hCTLA4Ig	Immunosuppressive agent	Up to 31.4 mg/L	Lee et al., 2007
		Der p 2-FIP-*fve* fusion protein	Immunomodulator and immunotherapeutic for allergies	10.5% TSP	Su et al., 2012
		hGM-CSF	Production of white cells	2% TSP	Kim et al., 2008b
		Human Serum Albumin	Treatment of hypoalbuminemia	up to 25 mg/L	Huang et al., 2005
		Human CTLS4Ig	Immunosupressive agent	Up to 31.4 mg/L	Lee et al., 2007; Kang et al., 2015
		Human Growth Hormone	Growth Hormone	Up to 120 mg/L	Kim et al., 2008a
		Granulocyte-Macrophage Colony- Stimulating Factor (GM-CSF)	Production of white cells	Up to 200 mg/L	Lee et al., 2007; Shin et al., 2011
Medicago cells	*Medicago truncatula* cv. Jemalong	EPO	Tissue protective		Pires et al., 2012
		Prostaglandin D_2_ Synthase	Clinical marker		Pires et al., 2014
Carrot cells	*Daucus carota*	Taliglucerase alfa	Gaucher disease		Shaaltiel et al., 2007
		PEGylated recombinant human acetylcholinesterase (PRX-105)	Biodefense program		Protalix Biotherapeutics (www.protalix.com)
		α1-antitrypsin (PRX-107)	Emphysema		Protalix Biotherapeutics (www.protalix.com)
Tomato cells	*Lycopersicon esculentum*	hGM-CSF	Immunosuppressive and immunomodulator	Up to 45 μg/L	Kwon et al., 2003a
Soybean cells	*Glycine max*	HBsAg	Vaccine against Hepatitis B	65 μg/g FW	Smith et al., 2002
Siberian Ginseng cells	*Acanthopanax senticosus*	Human lactoferrin	Immunosupressive and immunomodulator	0.2–2.3% TSP	Jo et al., 2006
Korean ginseng cells	*Panax ginseng*	Human lactoferrin	Immunosupressive and immunomodulator	3% TSP	Kwon S. Y. et al., 2003
Sweet Potato cells	*Ipomoea batatas*	Human lactoferrin	Immunosupressive and immunomodulator	3.2 μg/mg TSP	Min et al., 2006

*SN, supernatant; TSP, total soluble protein, FW, fresh weight*.

The production of recombinant proteins in plant cell suspension cultures can be achieved by transforming wild-type cells already in suspension and selecting those carrying a co-introduced marker gene, or by initiating cultures from transgenic plants. As with other fermenter-based systems, the scalability of plant cell cultures is limited by the bioreactor capacity but the product can be recovered from the medium allowing continuous production, or it can be directed to a specific internal compartment if this is more appropriate (Schillberg et al., 2013). Although inducible promoters in plants allow production to be divided into a growth phase and a production phase analogous to the inducible production systems used in bacteria and yeast, there is currently no counterpart of the amplification technologies used with mammalian cells so the product yields in plant cells are much lower—however, the assembly of an artificial system in plants is conceivable (BOX 3).

BOX 3 **THE CHO AMPLIFICATION SYSTEM AND CAN WE REPLICATE IT IN PLANTS?**

The CHO system is the most widely used mammalian cell line platform in the industry because it was the first to market and is therefore backed by years of cumulative experience and process optimization, and it is compatible with serum-free medium which reduces the potential bioburden (Wurm, 2004). Most of all it has a highly effective gene amplification system, paired with an unstable genome that facilitates amplification and other genetic changes (Cacciatore et al., 2010). This was discovered accidentally when rare individual CHO cells were shown to survive toxic concentrations of the drug methotrexate, which inhibits the enzyme dihydrofolate reductase (DHFR). The analysis of surviving cells showed that some carried point mutations conferring resistance but others contained multiple copies of the *dhfr* locus and produced enough of the enzyme to outcompete the inhibitor.

Stepwise selection at higher concentrations isolated cells with massively amplified *dhfr* gene arrays allowing survival at 10,000 times the normal toxic dose of methotrexate. The amplified genes were present as homogeneously staining regions within chromosomes or as small extra chromosomes called double minutes. Importantly, these arrays contain flanking regions as well as the *dhfr* gene itself so adjacent genes can also be amplified even if though they do not contribute to methotrexate-resistant phenotype (Cacciatore et al., 2010). The current industry CHO platform is based on the mutant cell line DG44 which lacks an endogenous *dhfr* gene. This cell line is transfected with a tandem *dhfr*-X construct, where X encodes the desired recombinant protein. Both genes are amplified under selection and the yield of the recombinant protein is boosted substantially. Many different amplifiable markers have been identified but only *dhfr*-methotrexate and glutamine synthase-methionine sulfoxamine are used for commercial pharmaceutical production.

There is no equivalent amplifiable marker system in plants although many of the markers which work in mammalian cells as amplifiable markers can be used for simple one-step selection in plants, including *dhfr* (Eichholtz et al., 1987). The failure of amplifiable selection therefore suggests that plants lack an intrinsic ability to generate massive arrays of small regions of the genome under selection, which indicates a difference in the capacity for homologous recombination. Such differences have been observed before, and explain the difference in gene targeting efficiency between mammals and plants (Puchta and Fauser, 2013). One potential solution to this issue is the use of extra chromosomal replicating vectors for amplification in plants, as reported by Regnard et al. (2010). Even without amplification, plant cells are moving toward parity with mammalian cells. For example, cell-specific production rates of 8 pg/cell/day have been reported for the monoclonal antibody M12 produced in tobacco BY-2 cells (Havenith et al., 2014) compared to typical production rates of 20–40 pg/cell/day for CHO cells carrying thousands of gene copies, showing that the difference between these systems is less than an order of magnitude.

Several platforms have emerged as contenders for a standardized production technology including cell suspension cultures derived from tobacco (*Nicotiana tabacum*), rice (*Oryza sativa*), and carrot (*Daucus carota*), which are the front runners today. The most widely used tobacco cell line is derived from the cultivar Bright Yellow 2 (BY-2). Tobacco BY-2 suspension cell cultures can multiply up to 100-fold within 7 days with a doubling time of 16–24 h under ideal conditions. The BY-2 cell line was developed in 1968 at the Hatano Tobacco Experimental Station, Japan Tobacco Company (Kato et al., 1972). The transformation of BY-2 cells using *A. tumefaciens* is highly efficient (Nagata et al., 1992) and therefore many different products have been successfully produced using this system (Table 1). One of the drawbacks of molecular farming in whole tobacco plants is that the leaves contain nicotine, but BY-2 cells do not produce significant amounts of this metabolite even when induced by jasmonates, and instead produce the related compound anatabine as well as low levels of other alkaloids (Shoji and Hashimoto, 2008).

Rice cell suspension cultures are used almost as widely as tobacco BY-2 cells due to the availability of the carbohydrate-sensitive α-amylase promoter system (RAmy3D) that works in synchronization with the fermentation cycle. This promoter is induced by sugar starvation, and gene expression can therefore be optimized by timing media exchanges so that cells are exposed to consecutive growth and production phases (Lee et al., 2007). Most rice varieties can be dedifferentiated but Japonica varieties appear more amenable than Indica varieties, such that callus can easily be produced from almost every part of the plant. Rice cell suspension cultures have a doubling time of 1.5–1.7 days (Trexler et al., 2005). Many pharmaceutical products have been expressed in rice cells (Table 1) and at least one major company has adopted rice cells as an industrial production platform, albeit for non-pharmaceutical-grade cosmetics ingredients and research reagents (Natural Bio-Materials, Jeollabuk-do, Korea; http://www.nbms.co.kr/).

Carrot cell lines can be derived from hypocotyl, epicotyl, or cotyledon tissues. The transformation of carrot cells can be achieved by co-cultivation with *A. tumefaciens*, particle bombardment or the electroporation of protoplasts (Rosales-Mendoza and Tello-Olea, 2015). The first plant-derived biopharmaceutical protein approved by the FDA for human use was taliglucerase alfa, produced in carrot cell suspension cultures by the Israeli company Protalix Biotherapeutics (http://www.protalix.com) and licensed by Pfizer (Table 1).

In addition to these three commercially-relevant platforms, several other plant species have been used to produce cell suspension cultures for molecular farming. The model legume *Medicago truncatula* (Abranches et al., 2005) is typically used for the analysis of secondary metabolism (Cook, 1999; Broeckling et al., 2005) but this species has been developed more recently for molecular farming because suspension cells can be derived from the mature leaf, root and seedling cotyledon, and transformation is highly efficient (Araujo et al., 2004). A cell line derived from this species was shown to achieve high recombinant protein yields (Pires et al., 2008) although only two biopharmaceutical products have been reported thus far (Pires et al., 2012, 2014). Other proteins have been produced in cell lines derived from tomato (*Solanum lycopersicum*), soybean (*Glycine max*), potato (*Solanum tuberosum*), sunflower (*Helianthus annus*), sweet potato (*Ipomoea batatas*), and medicinal plants such as Siberian ginseng (*Eleutherococcus senticosus*) and Korean ginseng (*Panax ginseng*). Biopharmaceuticals produced in these species are listed in Table 1.

## Progress and challenges

Although plant cells are relative newcomers in the commercial environment and the yields they achieve still lag behind those of microbes and mammalian cells, it is important to remember that the yields produced by microbes and mammalian cells have increased substantially during their 30 years as industrial platform leaders. These increases have been achieved incrementally by several routes, including strain optimization, genetic modification to improve production characteristics, medium optimization, and process engineering (e.g., bioreactor design and fermentation conditions). In contrast, plant cells have been used commercially for less than 10 years and already the improvements have been striking, mainly because the lessons learned during the development of microbial and mammalian cell platforms have been applied to plant cells comparatively much earlier in their history as a platform technology, and novel approaches adopted by the industry more recently have been used with plant cells immediately, and implemented during early process development. These include strategies such as high-throughput clone selection, medium and process optimization using statistical experimental designs (typically design-of-experiments approaches) and the application of in-process monitoring systems, known as process analytical technology (PAT). Given the lead time, it is clear that current industry-standard mammalian cell lines such as Chinese hamster ovary (CHO) cells will remain superior in terms of overall yields for some time to come, but plant cells are gaining ground due to the many advantageous properties they offer (Table 2). The main challenges that plant cells still face are the absence of a gene amplification system comparable to the systems used with CHO cells (BOX 3) and the convenience of handling issues that are important for GMP compliance, such as cryopreservation and cell banking (Eck and Keen, 2009; Mustafa et al., 2011).

**Table 2:** **Comparison among the available systems for biopharmaceutical production**.

**System**	**N-glycosylation ability**	**Contamination risk**	**Time to production**	**Scalability**	**Overall cost**
Plant cell suspensions	Yes	Very low	Medium	High	Medium
Whole plant systems	no terminal galactose or sialic acid; Core-xylose; different fucose linkage	Low	High	Very high	Low
Plant transient expression		Low	High	High	Low
Mammalian Cells	Yes but different potential sialic acid (NGNA) and alpha-Gal epitope both potential immunogenic	High	Medium	Medium	High

*Overall cost: Low, $20–100/g; Medium, $50–1000/g; High, $1000–10,000/g*.

## Specific challenges—cell clusters, growth characteristics, and culture heterogeneity

Almost all plant cell suspension cultures share one key property that sets them aside from microbial and mammalian cells—they do not grow as single cells but instead form clusters (Mavituna and Park, 1987; Nagata et al., 2013). Moreover, plant cells can grow significantly by elongation, increasing the volume and wet biomass without increasing the cell number. Both issues must be addressed by adopting specific methodologies. Although cell clusters can be advantageous, e.g., aggregation can be used as the basis for self-immobilization methods (Kieran et al., 1997; Kolewe et al., 2011), large cell clusters are generally undesirable because cells in the center may have limited oxygen and nutrient availability.

**Figure 1:**
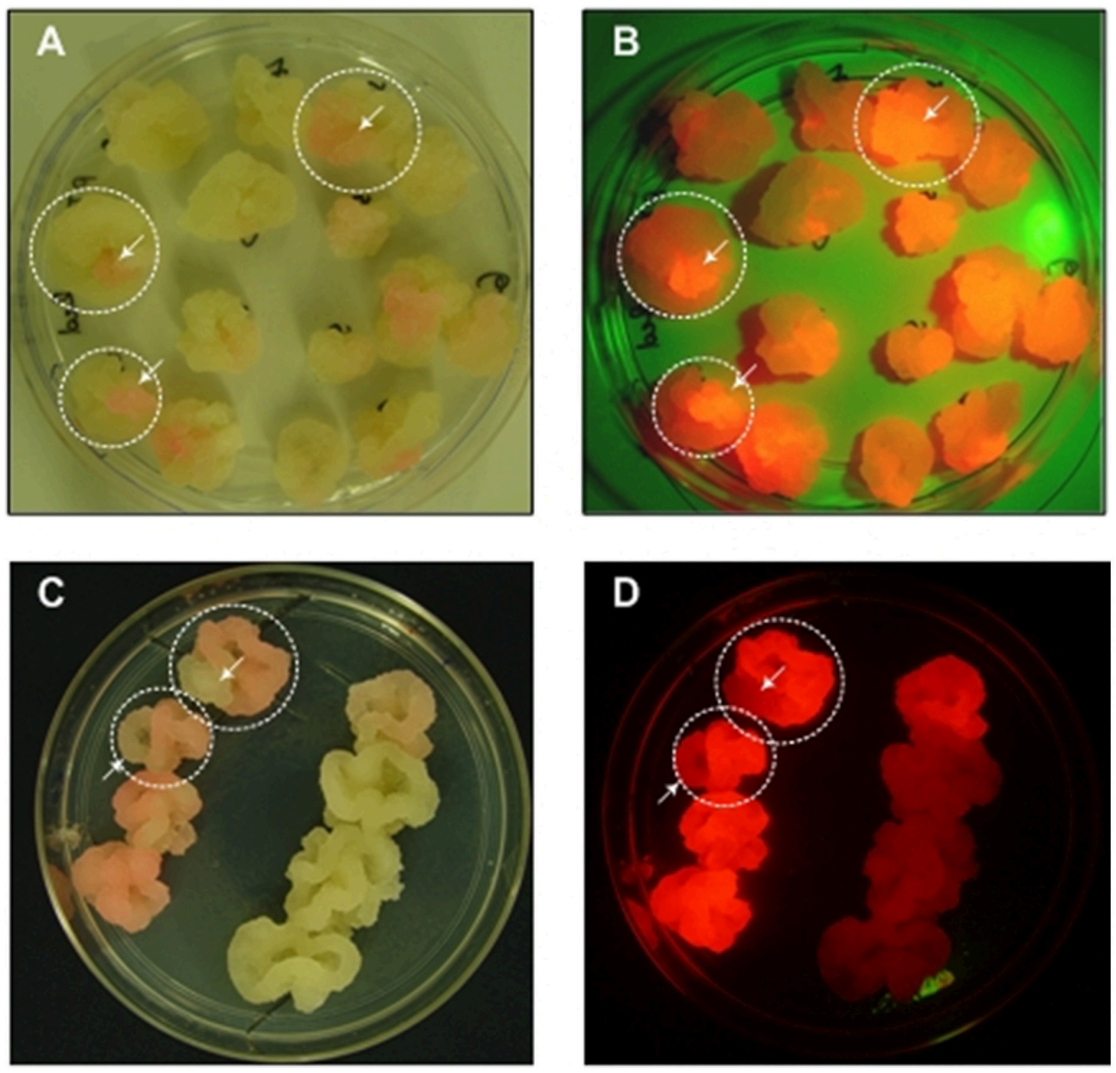
**Sectored callus cultures**. **(A,C)** Images were taken under normal white light, **(B,D)** Images were taken under green light with a red filter for the macroscopic visualization of DsRed fluorescence.

Cell clusters are also challenging during the generation of transgenic cell lines because monoclonal cultures cannot be generated by plating or limiting dilution. Cell suspension cultures generated *de novo* by the transformation of wild-type cells are always polyclonal because transformation is not 100% efficient and different cells can be transformed at different loci (Muller et al., 1996; Nocarova and Fischer, 2009). Even cell lines derived from transgenic callus are rarely monoclonal because the callus tissue may be chimeric. In both cases, the resulting transgenic cell lines can also undergo somaclonal variation, generating cell populations with heterogeneous expression levels (James and Lee, 2006). Therefore, even if an advanced technology such as the CRISPR/Cas9 system is used to specify a targeted integration site, one or more rounds of screening and selection is still necessary to identify and isolate the most productive cells to seed monoclonal production lines. Screening can be carried out at the callus stage and the use of fluorescent marker proteins facilitates the identification of chimeric callus tissue, allowing the selection of cell material for sequential rounds of sub culturing (Figure 1). An alternative is the preparation of protoplasts with subsequent selection by flow cytometry, although single protoplasts are fragile and plating on feeder cells is often required. Recently, flow sorting has been used to separate the most productive cells from a heterogeneous tobacco BY-2 cell culture producing a full-length human antibody, by selecting the co-expressed fluorescent marker protein DsRed located on the same T-DNA (Kirchhoff et al., 2012). Using a feeder strategy, single protoplasts selected by flow cytometry were regenerated into stable monoclonal cell lines with homogeneous DsRed fluorescence and antibody yields up to 13-fold higher than the parent culture.

The productivity and growth characteristics of cell lines at the callus stage and in suspension are often unrelated thus raising additional challenges. Although fluorescent marker proteins can be used to screen callus tissue, it is good practice to continue screening the suspension cells under realistic production conditions to ensure a compromise between protein production and cell growth rates. CHO cell lines also display idiosyncratic behavior with respect to stability, media requirements and other process performance parameters. The current industry solution is to screen a sufficiently large number of clones under rigorous selection criteria to ensure that high-performance clones are identified, and this strategy is equally applicable to plant cell suspension cultures.

Because plant cells are large and tend to grow in clusters, it can be difficult to determine accurate cell densities. Plant cells are 50–200 μM in length and range in morphology from spherical to cylindrical depending on the growth phase. Cells in the exponential growth phase undergoing rapid division are spherical or elliptical, with a length of 50–100 μm, whereas those at the end of the exponential growth phase grow mainly by elongation and tend to be more cylindrical, with a length of up to 200 μm (Mavituna and Park, 1987; Holland et al., 2013). Aggregation occurs when daughter cells fail to separate after cell division, and is promoted by extracellular polysaccharides. The tendency for form clumps varies between cell lines and depends on the age of the cells and the growth conditions. Cell counting is the most precise method to establish cell density but it becomes more difficult when the cells clump together. Alternative methods such as the measurement of turbidity or light scattering are also unsuitable due to the size of the clumps. Therefore, the density of plant cell suspension cultures is often determined by measuring the packed cell volume or wet cell weight after gentle centrifugation and aspiration of the supernatant. Alternatively, the pellet can be dried and cell density can be extrapolated from the dry weight. However, these are invasive and destructive off-line procedures. The use of non-invasive radio frequency impedance spectroscopy (RFIS) offers significant benefits because it can achieve continuous in-line real-time measurement suitable for PAT. Although RFIS measures the volume of viable cells, this parameter correlates well with the packed cell volume, wet cell weight, and dry biomass weight. Continuous measurement can also pinpoint the transition from cell division to cell elongation (Holland et al., 2013).

## Specific challenges—medium optimization

The productivity of cell suspension cultures can be improved by optimizing the expression construct and by selecting highly-productive monoclonal cultures, but it is also necessary to optimize the culture conditions starting with the growth medium (Schillberg et al., 2013). Unlike whole plants, cell suspension cultures are not phototrophic so they require a carbon source. Plant cell media therefore usually contain sucrose, inorganic salts, vitamins, plant hormones and water, and a wide range of different media are commercially available depending on the species, growth characteristics, and purpose of the cultivation (Fawcett, 1954; Murashige and Skoog, 1962; Gamborg et al., 1968). In many cases, the growth medium is a variant of the MS recipe developed by Murashige and Skoog (1962), which provides nitrogen as a mixture of nitrate and ammonium salts. However, the addition of more nitrogen to MS medium can improve the productivity of BY-2 cells by up to 150-fold in the stationary phase, ultimately improving yields of recombinant proteins by up to 20-fold (Holland et al., 2010; Ullisch et al., 2012). In contrast to CHO cells, where medium optimization needs to be done for each product and cell line on a case-by-case basis (Wurm, 2004), for plant cell cultures it appears to similarly benefit all products (Holland et al., 2010; Ullisch et al., 2012). This was one catalyst for the introduction of statistical experimental designs that can simultaneously test the impact of varying several different medium components simultaneously, as well as other conditions such as pH, temperature and aeration rate. Accordingly, product-specific medium optimization achieved a five-fold increase in the yield of a recombinant antibody produced by tobacco BY-2 cells following the application of a statistical experimental design (Vasilev et al., 2013). The impact of changes in medium composition during fermentation, and the introduction of compensatory in-line adjustments, has also boosted product yields substantially. For example, a respiration activity monitoring system (RAMOS) revealed metabolic changes in cultivated BY-2 cells caused by ammonia depletion, and the replacement of this missing ammonia resulted in a 100% increase in product yields (Ullisch et al., 2012).

## Specific challenges—protein degradation

Degradation caused by intracellular and extracellular proteases reduces the yield and quality of biopharmaceuticals produced in plant cells, and extra purification steps are required to remove degradation products. Extracellular degradation can be avoided by targeting the protein to accumulate within an intracellular compartment, and the endoplasmic reticulum (ER) is often used for this purpose because complex proteins fold efficiently and accumulate to higher levels than those secreted to the apoplast, i.e., the space under the cell wall (Twyman et al., 2013). However, the benefits of intracellular accumulation must be balanced against two drawbacks—the need to extract the protein by breaking the cell, which releases more contaminants (including proteases) during downstream processing (Buyel et al., 2015), and the impact on glycosylation, which is discussed in the next section. A better approach is to allow secretion but to counter the effect of proteases directly. Plants produce hundreds of proteases and it is not always possible to identify which is responsible for degrading a recombinant protein, particularly because different products are susceptible to different protease classes (Mandal et al., 2010, 2014; Navarre et al., 2012; Niemer et al., 2014). If a particular protease can be identified then it may be possible to knock out the corresponding gene or co-express a protease inhibitor to prevent product degradation (Kim et al., 2008b; Benchabane et al., 2009). Decoy proteins such as gelatin or bovine serum albumin can also be added to the medium but proteinaceous additives from animal sources must be evaluated carefully because they pose a risk of contamination with prions, thus compromising the economic and regulatory advantages of plant cells (James et al., 2000; Baur et al., 2005). Non-protein additives such as polyvinylpyrrolidone (Magnuson et al., 1996; LaCount et al., 1997), Pluronic F-68 and polyethylene glycol (Lee and Kim, 2002) can also reduce the damage caused by proteases but may be difficult to remove in subsequent processing steps (Baur et al., 2005). Osmotic stress has also been proposed to increase product accumulation, although this may inhibit cell growth so the timing of application must be optimized carefully (Tsoi and Doran, 2002; Soderquist and Lee, 2005). Medium optimization and process control are promising tools to avoid protein degradation during production—for example, a balanced supply of nitrogen not only dramatically increases the amount of a secreted antibody but also stabilizes the secreted product toward the end of the cultivation process (Holland et al., 2010).

## Specific challenges—plant glycans

The early steps of protein glycosylation in plants and mammals are identical, but once a nascent protein moves from the ER to the Golgi apparatus subtle differences in the oligosaccharide structures begin to appear. Plant glycoproteins tend to contain core α1,3-fucose (rather than core α1,6-fucose which is present in mammals) and core β1,2-xylose, whereas mammalian glycoproteins contain β1,4-galactose and terminal sialic acid residues that are not present in plants (Gomord et al., 2010). Initially there was concern that plant glycans could be immunogenic in humans and much effort was expended to ensure that plant glycans were avoided. This involved either targeting the proteins to be retained in the ER resulting in generic high-mannose glycans, or engineering plant lines in which the glycosylation pathway was modified to abolish the enzymes responsible for plant glycans and, in some cases, introduce enzymes that produced human-like glycans instead (Castilho and Steinkellner, 2012; Bosch et al., 2013). The glycan panic has since abated given the lack of evidence that plant glycans are harmful in humans (Gomord et al., 2010). The first-in-class Protalix drug taliglucerase alfa (Table 1) contains the aforementioned core α1,3-fucose and β1,2-xylose residues but no adverse effects have been reported in clinical trials or post-market use (Tekoah et al., 2015).

Although plant glycans can affect the properties of recombinant proteins, including stability and functionality, in some cases the plant-derived version is superior—not biosimilar but bio-better. In the case of taliglucerase alfa, targeting the protein to the vacuole of carrot cells exposes terminal mannose residues that are required for the efficient uptake of the enzyme into macrophages by mannose receptors. The equivalent protein produced in CHO cells (imiglucerase, marketed as Cerezyme®) has terminal sialic acid residues that prevent uptake, and these must be enzymatically removed *in vitro* during processing, which increases the production costs dramatically. The comparison between taliglucerase alfa and imiglucerase also highlights the safety advantages of plant cells. The production of imiglucerase in CHO cells by Genzyme was shut down for a significant time due to viral contamination in the production plant (Ema, 2009). This resulted in an acute shortage of the product because the plant was responsible for ~20% of the global supply at that time (Hollak et al., 2010).

## Specific challenges—upstream processing strategies

Plant cell cultures are often successful in the laboratory because they can be grown in well-aerated shake flasks and the products can be extracted in small volumes of buffer, allowing the use of protease inhibitors and other expensive additives that cannot be used at the process scale. Cell line selection and optimization also tends to be carried out using small flasks or even microtiter plates, so scaling up production is a significant challenge (Fischer et al., 2015). Plant cell cultures have been cultivated in many different bioreactors, like stirred tanks reactors (STR's; Hooker et al., 1990; Doran, 1999; Trexler et al., 2002), wave reactors (Eibl and Eibl, 2006), wave and undertow (Terrier et al., 2007), bubble column (Terrier et al., 2007), single use bubble column reactor (Shaaltiel et al., 2007), air-life reactors (Wen Su et al., 1996), membrane reactors (McDonald et al., 2005), and rotation drum reactors (Tanaka et al., 1983). The homogeneous nature of plant cell suspension cultures requires a fermentation broth similar to that used for microbes and mammalian cells, which is fed with nutrients and oxygen and mixed to achieve an even distribution (Hellwig et al., 2004). In many reports, stirred-tank bioreactors with large impellers and a ring sparger have been used to reduce shear stress, delivering maximum biomass values of 60–70% packed cell volume. In the context of cell suspension cultures the bioreactor must be matched to the production line to synergize with its biological characteristics, e.g., growth rate, morphology, aggregation tendency, shear sensitivity, oxygen demand, and rheological properties. The relative merits of the different reactor designs have been intensively discussed and reviewed elsewhere (Xu et al., 2010; Huang and McDonald, 2012).

The cultivations in the different reactor designs covering a range of volumes and offering three main fermentation strategies: batch, fed-batch and continuous processes. The batch fermentation is the simplest and most commonly used process. A batch fermentation is filled with medium, inoculated and after inoculation the reactor is a closed system except for a few additives like oxygen and base/acid for controlling the pH-value. The cell culture undergoes a lag, exponential and stationary growth phase and cell growth generally occurs under varying and sometimes unfavorable conditions. A more advance fermentation modes is the fed-batch fermentation, which starts with a classical batch phase and once certain conditions are reached the feed is started, i.e., additional nutrients are provided. In fed-batch also several culture parameters are changing and the cells undergo classical growth phases. Furthermore, batch and fed-batch fermentations suffer from low running-to-set-up-times ratio (preparation, sterilization before the cultivation, and cleaning afterwards), which can be compensated by investments of men-power and infrastructure. To overcome low running-to-set-up-times different continuous fermentation strategies for plant cell cultures has been developed. Classical continuous fermentations strategies are perfusion and chemostat processes. In perfusion processes the cells in the reactor are supported with continuous feed of fresh media and cell free fermentation broth is constantly being removed in the same volume. Perfusion fermentation have successfully realized with plant cell cultures (Su and Arias, 2003; De Dobbeleer et al., 2006). In a chemostat fermentation the culture is also being supported by a continuous feed of fresh medium but in this case the same volume of fermentation broth which is being removed also contains cells, thus in a perfusion the cell density is increasing while in a chemostat the cell density stays constant. A chemostat cultivation can run over a long time period and the cells are maintained at the exponential growth phase, which make this strategy attractive for large scale production (Miller et al., 1968; van Gulik et al., 2001). Despite the ideal characteristics of the continuous bioreactor, the process itself is sensitive and subjected to influence from various factors such as risks of contaminations, genetic instability, and changes in the biotic phase of the bioreactor. To avoid these semi-continuous fermentation strategies have been developed, where a fraction of the fermentation broth is removed once and replaced by fresh medium (Hogue et al., 1990; Huang et al., 2010). The merits of the different cultivation strategies is summarized and discussed intensively from Xu et al. (2011). Some recent examples for large scale cultivations are the manufacture of antibodies in tobacco BY-2 cells, which has been scaled up from shake flasks to 200-L disposable bioreactors without loss of yield (Raven et al., 2015). Scaling up from 50-ml shake flasks to 600-L bioreactors (a factor of 12,000) has also been achieved without any impact on growth characteristics (Reuter et al., 2014). Taliglucerase alfa and other products in the Protalix pipeline are produced in carrot cells cultivated in bubble column-type bioreactors fitted with disposable polyethylene bags (Shaaltiel et al., 2007; Tekoah et al., 2015).

## Specifc challenges—cell banking

To meet the regulatory requirements it will be necessary to ensure cell line stability over the entire production process time. Many cell cultures are maintained by a weekly sub culturing routine and are stable over long time period, nevertheless there are only few early studies on long term production stability done (Gao et al., 1991; Sierra et al., 1992; Kirchhoff et al., 2012). Cell banking for the supply of well-defined starting material and a routine procedure for the cryopreservation of plant cells is one key feature to enable plant suspension culture as a biopharmaceutical production platform. The first successful cryo preservations of plant cell cultures have been reported in the late 1960s and 1970s (Quatrano, 1968; Nag and Street, 1973). Since then several protocols have been developed, e.g., for particular cell lines like BY-2 cells and tobacco cell lines (Menges and Murray, 2004; Schmale et al., 2006) or arabidopsis cells (Menges and Murray, 2004; Ogawa et al., 2008). Although different techniques have been published, including desiccation (Nitzsche, 1980), vitrification (Uragami et al., 1989) or encapsulation-dehydration cryopreservation (Bonnart and Volk, 2010), there is no protocol that can generally be applied to cell suspension of all plant species and all protocols need to be optimized on a case-by-case basis. Only few protocols have been verified for different cell species (Ogawa et al., 2012).

## Conclusions

In the near future, plant cell suspension cultures will most certainly become the preferred choice among plant-based systems for the production of high-value recombinant proteins, because they combine the advantages of all other systems. Although plant cells have been overshadowed by whole-plant platforms, this trend has been inverted following the approval of taliglucerase alfa for use in human adults in 2012 and then for pediatric use in 2014 (Tekoah et al., 2015). This has opened the way to the full acceptance of this technology, and several other products are now undergoing clinical trials and are expected to reach the market in the near future. The significant number of drugs that are now coming off patent will contribute to this market expansion.

Plant-based systems still face one major bottleneck that needs to be overcome—their lower yields compared to mammalian cell cultures. This partly reflects the much more recent emergence of plant cells as a competitive platform, so there has been less investment thus far in strain, medium and process optimization compared to mammalian cells. However, there are many researchers currently working to address this challenge, and several recent reports discussed herein have made breakthroughs in the development of robust upstream production and downstream processing strategies. These developments include medium optimization, process engineering, statistical experimental designs, scale-up/scale-down models, and process analytical technologies. Overall, these optimization procedures will lead to higher yields and will put plant cell cultures back into the spotlight. Other factors that will also contribute to the success of plant cells include the straightforward compliance with GMP compared with whole plants, and the better public acceptance of biopharmaceuticals produced in cultivated cells than GM plants.

## Author contributions

All authors listed, have made substantial, direct and intellectual contribution to the work, and approved it for publication. All authors contributed equally in the Introduction, Progress and challenges and conclusion parts. RS and RA contributed in the specific Challenges: Cell clusters, growth characteristics, and culture heterogeneity, protein degradation and in making and designing the tables. TH, MS, and RF contributed in the specific challenges: Medium optimization, plant glycans, upstream processing and cell banking.

## Funding

This work was funded by Fundação para a Ciência e Tecnologia (FCT, Portugal) through grants ERA-IB/0001/2012, PTDC/BIA-PLA/2411/2012, and UID/Multi/04551/2013 and by the Fraunhofer Future Foundation project “Innovative technologies to manufacture ground-breaking biopharmaceutical products in microbes and plants” (125-300004).

### Conflict of interest statement

The authors declare that the research was conducted in the absence of any commercial or financial relationships that could be construed as a potential conflict of interest.
